# *Lactobacillus*‐Loaded Bioadhesive Hydrogel with Prolonged Retention and Microenvironmental Remodeling for Endometrial Repair and Fertility Restoration

**DOI:** 10.1002/advs.77229

**Published:** 2026-08-21

**Authors:** Lifa Chen, Hui Zhou, Yifei Li, Yi Liang, Yifan Zhang, Huiyu Huang, Qiuxian Xie, Youchen Tang, Huixia Ye, Yu Zhang

**Affiliations:** ^1^ Department of Gynecology The Third Affiliated Hospital Sun Yat‐sen University Guangzhou China; ^2^ School of Chemistry Key Laboratory for Polymeric Composite & Functional Materials of Ministry of Education Sun Yat‐sen University Guangzhou China; ^3^ Colorectal Surgery Unit III Guangdong Institute of Gastroenterology Biomedical Innovation Center Key Laboratory of Human Microbiome and Chronic Diseases Guangdong Provincial Key Laboratory of Colorectal and Pelvic Floor Diseases Sun Yat‐sen University Ministry of Education The Sixth Affiliated Hospital Sun Yat‐sen University Guangzhou China; ^4^ Department of Gynecology Chaozhou Central Hospital Chaozhou China; ^5^ The Eighth Affiliated Hospital Sun Yat‐sen University Shenzhen China

**Keywords:** adhesive hydrogel, endometrial injury, fertility restoration, *L. johnsonii*, microenvironment modulation

## Abstract

Endometrial injury often causes inadequate regeneration and infertility, yet current hydrogels fail to meet the dual demands of sustained intrauterine retention and effective pro‐regeneration abilities for endometrial repair. Herein, we have developed a bioactive adhesive hydrogel (LAT@L) by facilely integrating three natural medicinal molecules, α‐lipoic acid (LA), L‐arginine (Arg), and tannic acid (TA) with *Lactobacillus johnsonii* (*L. johnsonii*), which exhibits long‐term intrauterine retention and microenvironment modulation to promote endometrial repair. Through the spontaneous formation of covalent bonds, hydrogen bonds, and electrostatic interactions among LA, TA, and Arg, a LAT@L hydrogel with a stable network can be prepared via one‐pot mixing. Meanwhile, LAT@L hydrogel with adhesive functional groups can also firmly adhere to the uterine surfaces via those multiple interactions. The excellent cohesion and adhesion synergistically enable an intrauterine retention exceeding 14 days. Concurrently, leveraging the anti‐inflammatory and antioxidant properties of LA, TA, and Arg, together with the microbiota‐modulating activity of *L. johnsonii*, LAT@L hydrogel effectively reprograms the local microenvironment into a pro‐regenerative state. Benefiting from the prolonged intrauterine retention and multifaceted microenvironmental modulation, our LAT@L hydrogel significantly enhances endometrial repair and promotes the recovery of fertility in a rat model of endometrial injury, holding substantial potential for clinical translation.

## Graphical abstract

A bioactive adhesive hydrogel capable of long‐term uterine retention (>14 days) and local microenvironment modulation is developed by straightforward integration of α‐lipoic acid, L‐arginine, tannic acid, and *Lactobacillus johnsonii*, which can markedly enhance endometrial repair and restore fertility in a rat model of endometrial injury.

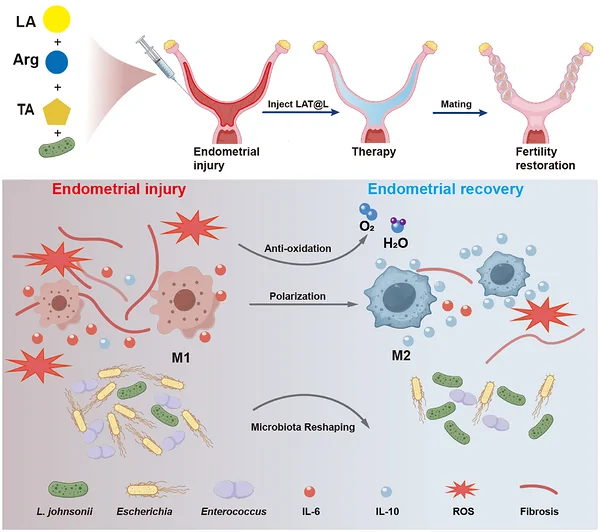

## 1 Introduction

Infertility represents a substantial public health concern in contemporary society, affecting approximately one in six couples who encounter difficulties related to reproductive health [1, 2, 3]. The uterine factor of infertility, primarily arising from damage to the endometrium, is becoming more prevalent, with approximately 6% of women undergoing uterine repair procedures [2]. The endometrium, a critical site for embryo implantation, is anatomically divided into the functional layer and the basal layer, wherein the functional layer plays a pivotal role in the establishment of pregnancy [3, 4]. However, various intrauterine procedures along with uterine inflammation can disrupt the structural integrity of the endometrium [5]. This disruption often results in inadequate regeneration of the functional layer, ultimately leading to failure in embryo implantation. Therefore, the treatment of endometrial injury must focus not only on restoring the compromised uterine architecture but also on promoting endometrial regeneration and enhancing endometrial receptivity.

Currently, the treatment of endometrial injury can be achieved by using hydrogels as a physical barrier, a strategy underpinned by their favorable biodegradability and biocompatibility. However, conventional hydrogels generally struggle to meet the dual demands of sustained intrauterine retention and effective pro‐regenerative bioactivity necessary for endometrial repair [6, 7]. First, the inadequate interfacial adhesion to tissue renders these materials vulnerable to displacement and detachment under the shear forces exerted by intrauterine fluids, significantly reducing their effective residence time [8, 9, 10]. Second, the mechanically unstable network structure resulting from low crosslinking density is susceptible to disintegration and structural collapse due to swelling in the humid uterine environment, thereby further undermining their physical barrier function [11]. Moreover, the post‐traumatic endometrial microenvironment is pathologically defined by sustained inflammatory infiltration, increased oxidative stress, and dysregulated macrophage polarization, all of which contribute to the advancement of fibrosis [12, 13, 14, 15]. Hydrogels that lack specific bioactive functions merely serve as passive space fillers and are ineffective at interrupting these pathological processes, thereby failing to reverse fibrosis or promote the functional restoration of the damaged endometrium. Therefore, the primary challenge that urgently requires resolution in the field of endometrial injury treatment is the development of a hydrogel system that offers prolonged tissue residence and microenvironmental regulation.

In recent years, living bacterial hydrogels have garnered significant attention as an innovative platform for bioactive materials [16]. Unlike traditional inert hydrogels, these living systems possess the capability to continuously secrete beneficial metabolites, actively modulate the local immune microenvironment, inhibit pathogenic colonization, and consistently deliver pro‐regenerative signals, demonstrating distinct advantages in facilitating tissue repair [17, 18]. In the context of uterine repair, growing evidence highlights the pivotal role of the uterine microbiota in sustaining endometrial homeostasis, with dysbiosis closely linked to chronic endometritis and fibrosis [19, 20, 21]. Consequently, *Lactobacillus*‐based living hydrogels show considerable potential for reestablishing a healthy microbial ecosystem while concurrently modulating inflammation.

**FIGURE 1:**
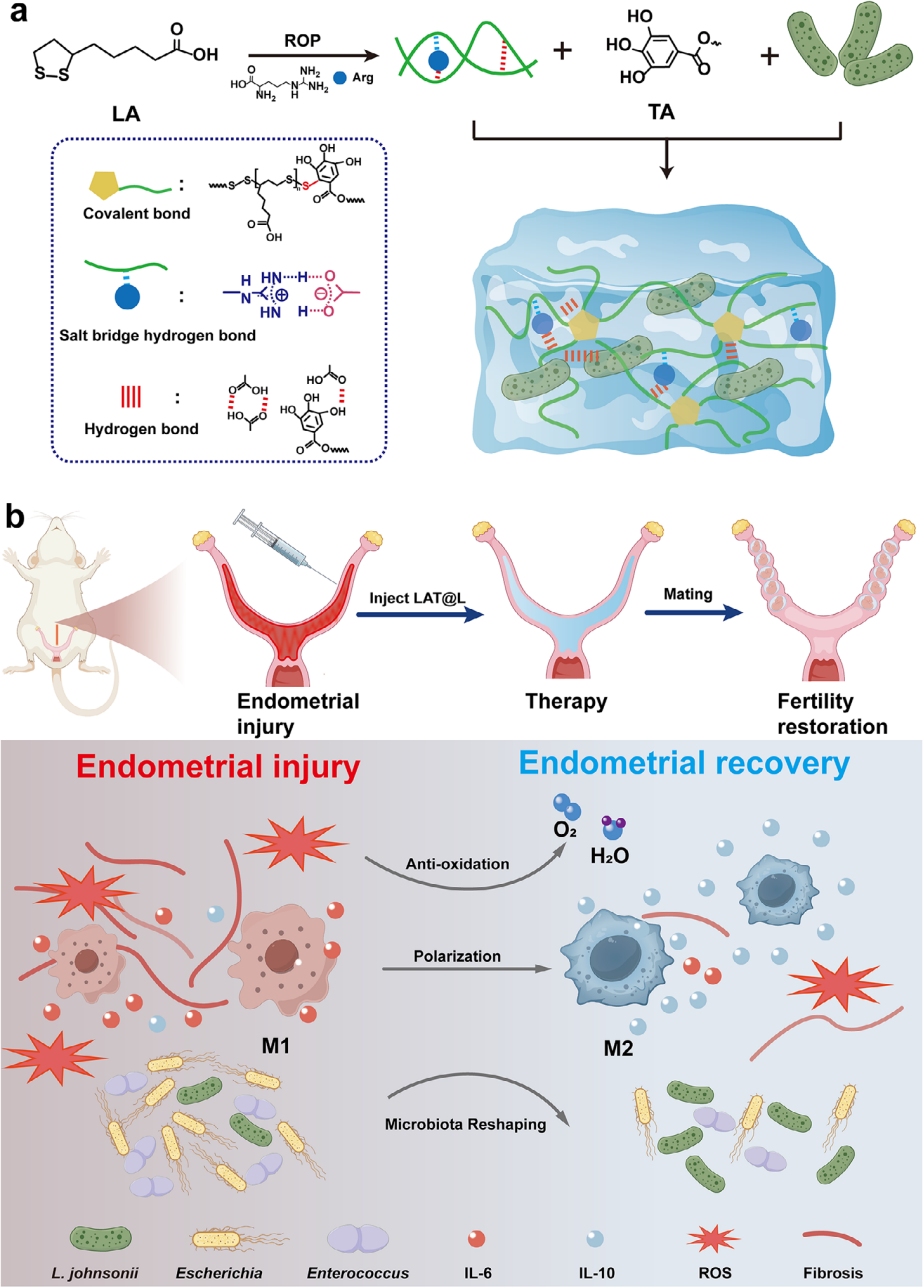
Schematic diagram of the preparation of LAT@L hydrogel and its therapeutic performance for endometrial injury. (a) The synthetic scheme of LAT@L hydrogel. (b) LAT@L hydrogel exhibits significant efficacy in endometrial repair and fertility restoration by reducing the level of oxidative stress, improving the inflammatory microenvironment at the injury site, and reestablishing uterine ecological balance.

Herein, we have successfully developed a bioactive adhesive hydrogel (denoted as LAT@L) by facilely integrating three natural medicinal molecules, *α*‐lipoic acid (LA), L‐arginine (Arg), and tannic acid (TA) with *Lactobacillus johnsonii* (*L. johnsonii*), which exhibits long‐term retention in the uterine cavity and the ability to modulate the local microenvironment to promote endometrial repair (Figure 1). Specifically, the alkaline property of Arg, an amino acid known for its pro‐neovascularization function, facilitates the deprotonation of natural antioxidant LA to form an LA salt with an enhanced aqueous solubility, which further promotes the ring‐opening polymerization of LA into poly(lipoic acid) (polyLA) chains. The anti‐inflammatory and antioxidant TA reacts with these chains to form covalent linkages, while the guanidine groups of Arg establish strong salt‐bridge hydrogen bonds with the carboxyl groups of polyLA, thereby building a stable hydrogel network. During gelation, *L. johnsonii* is encapsulated within the network, yielding the targeted LAT@L hydrogel. Owing to the residual disulfide bonds, carboxyl groups, and polyphenolic moieties, LAT@L hydrogel can adhere to tissue surfaces through multiple covalent bonds, hydrogen bonds, and electrostatic interactions. The excellent cohesion and adhesion resulting from these synergistic interactions endow LAT@L hydrogel with a long‐term retention capacity in the uterine cavity exceeding 14 days. Meanwhile, leveraging the inherent anti‐inflammatory and antioxidant effects of LA, TA, and Arg, as well as the beneficial remodeling effect of *L. johnsonii* on the uterine microbial community, LAT@L hydrogel can reshape the local microenvironment favorable for tissue regeneration. Consequently, LAT@L hydrogel markedly enhances endometrial repair and facilitates fertility recovery in a rat model of endometrial injury. This work may provide a new avenue for sophisticated biomaterials in endometrial repair and has important implications for the development of multifunctional biological materials.

## 2 Results and Discussion

### 2.1 Synthesis and Structural Characterization

The injured endometrium faces a complex microenvironment characterized by inflammation and oxidative stress. To this end, three natural medicinal molecules with related physiological functions are selected to construct an injectable bioactive hydrogel network through chemical bonding and supramolecular interactions (Figure S1). In detail, LA is an endogenous biomolecule with natural antioxidant function and is commonly used to construct biological hydrogels through spontaneous ring‐opening polymerization under mild conditions [22, 23]. Arg, a naturally occurring alkaline amino acid crucial for wound healing, is employed to enhance the solubility of LA in water and to facilitate preliminary cross‐linking between polyLA chains via salt‐bridge hydrogen bonds [24, 25]. The anti‐inflammatory and antioxidant TA covalently crosslinks to the polyLA chains by thiyl radical‐polyphenol Michael addition [23]. Through these multiple synergistic interactions among LA, Arg, and TA, the corresponding LAT hydrogel can be prepared by sequential mixing under ambient conditions (Figure S2). During gelation, *L. johnsonii* is encapsulated within the network, resulting in a LAT@L hydrogel.

**FIGURE 2:**
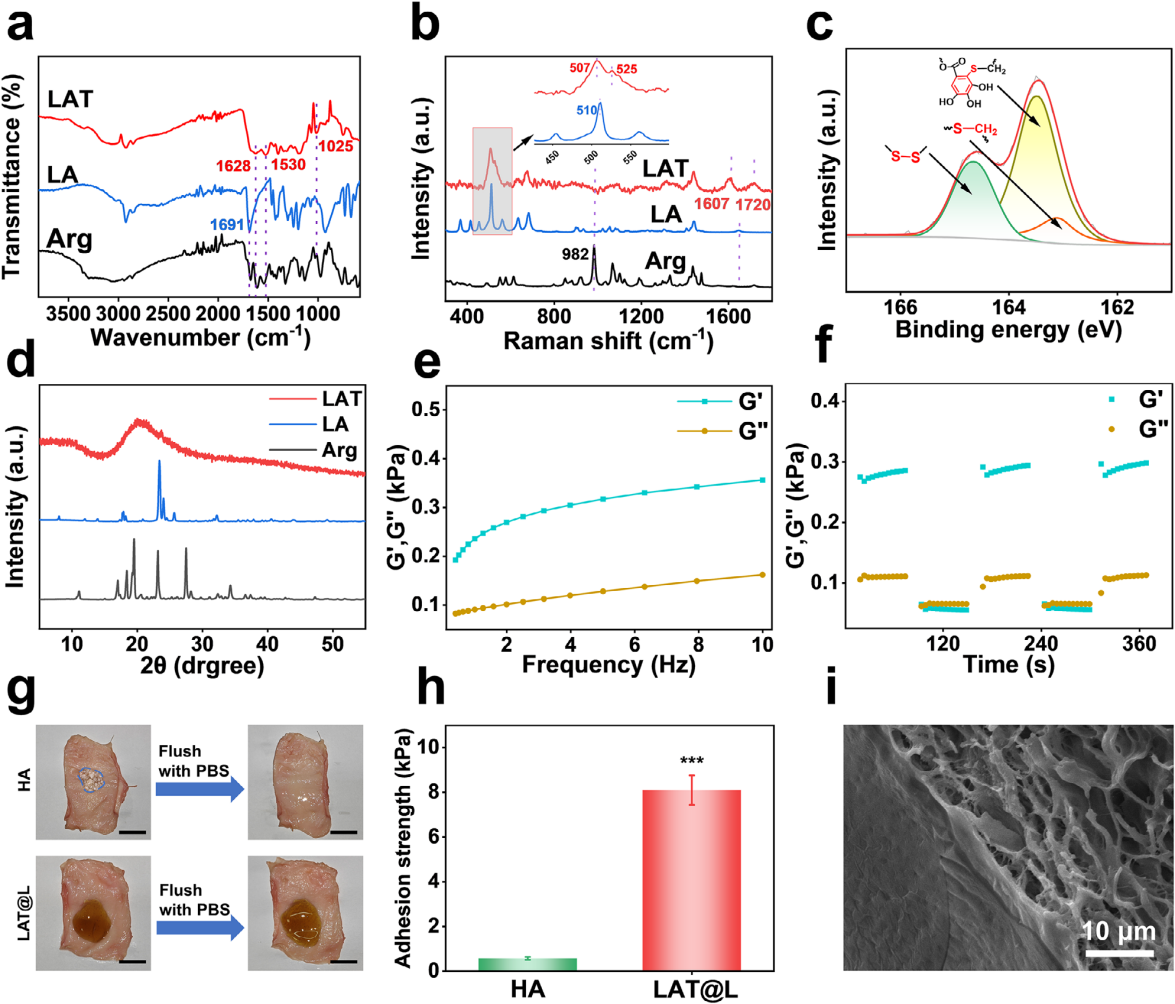
(a) FTIR and (b) Raman spectra of LAT hydrogel, LA, and Arg. (c) S2p XPS high‐resolution scanning spectrum of LAT hydrogel. (d) XRD patterns of LAT hydrogel, LA, and Arg. (e) Oscillatory frequency scanning and (f) cyclic amplitude scanning (at 1% and 400% strain) curves of LAT@L hydrogel. (g) Digital photos of HA and LAT@L hydrogels on the damaged porcine endometrial surface before and after flushing under water flow (scale bars: 1 cm). (h) Adhesion strength of HA and LAT@L hydrogels. (i) SEM image of LAT@L hydrogel adhesion interface with damaged porcine endometrium. All data are presented as mean ± SD (*n* = 3), and intergroup differences were evaluated using one‐way ANOVA followed by Tukey's post‐test. Statistical significance was defined as ^***^ *p* < 0.001.

The FTIR spectra (Figure 2a) reveal that, unlike LA, LAT hydrogel shows a new peak at 1530 cm^−1^, indicating the presence of carboxylate salts and suggesting partial deprotonation of LA due to the alkalinity of Arg. Moreover, the carboxyl peak of LA in LAT hydrogel system shifts from 1691 to 1628 cm^−1^, implying the formation of strong ionic hydrogen bonds between the carboxyl groups of LA and the carboxylate groups of LA‐salts, as well as the formation of salt‐bridge hydrogen bonds between the carboxyl groups of LA and the guanidine groups of Arg [24]. Raman spectroscopy shows that the LA monomer's disulfide bond peak at 510 cm^−^^1^ splits into two peaks at 507 and 525 cm^−^^1^ in LAT hydrogel (Figure 2b), indicating the ring‐opening polymerization of a disulfide‐containing five‐membered ring [26, 27, 28]. X‐ray photoelectron spectroscopy (XPS) was further conducted to analyze the chemical structures. As shown in Figure S3, LAT hydrogel exhibits a carboxyl peak at 288.8 eV in the C1s spectrum, which is shifted to lower binding energy relative to that of LA, and an additional peak at 401.3 eV in the N1s spectrum, assigned to deprotonated carboxylic acid carbon and protonated nitrogen [29, 30]. These observations substantiate the presence of a robust ionic hydrogen bond between the carboxyl group of LA and the carboxylate of LA salt, as well as a salt‐bridge hydrogen bond between the carboxyl group of LA and the guanidinium group of Arg within the hydrogel matrix. The S2p spectrum of LAT hydrogel shows three peaks: disulfide at 164.6 eV, aliphatic organosulfur at 163.0 eV, and thiophenol at 163.5 eV (Figure 2c), while LA only exhibits disulfide and aliphatic organosulfur peaks (Figure S3d). This indicates that the polyphenol groups in TA play an important role as scavengers for thiyl radicals, leading to chemical crosslinking during the hydrogel formation. X‐ray diffraction (XRD) analysis shows that the characteristic crystalline peaks of LA and Arg disappear in LAT hydrogel (Figure 2d), indicating a transformation from an ordered structure to an amorphous structure and molecular‐level combination. Collectively, these results demonstrate the successful construction of LAT hydrogel via multiple interactions.

During the cross‐linking process of LAT hydrogel, a phosphate‐buffered saline (PBS) suspension containing *L. johnsonii* was incorporated. The resulting LAT@L hydrogel retains a 3D network structure, effectively encapsulating *L. johnsonii* and ensuring its stable retention within the interconnected pores (Figure S4). The cumulative release of *L. johnsonii* reaches 82.3% ± 3.2% at 72 h (Figure S5), which suggests successful encapsulation and efficient release. Rheological analysis shows a stable gel network, with the storage modulus (*G′*) consistently exceeding the loss modulus (*G′′*) at 25°C (Figure 2e). Strain amplitude scanning at 0.1 Hz and 25°C reveals a gel‐to‐sol transition at 398.7% shear strain, where *G′* fell below *G′′* (Figure S6a). The cyclic amplitude scanning of LAT hydrogel in Figure 2f demonstrates that at low strain (1%), *G′* is greater than *G′′*, indicating its gel state; at high strain (400%), *G′* decreases significantly and the gel network structure is destroyed (*G′*< *G′′*). When the strain changes to low strain (1%) again, *G′* can quickly return to the initial state. Hence, LAT@L hydrogel demonstrates shear‐thinning behavior (Figure S6b), self‐healing ability (Figure S7a), and injectability (Figure S7b). These properties are essential for hydrogels in practical applications [31]. They ensure minimal resistance during injection through narrow catheters and enable the material to self‐reconstitute and adapt to the morphology of the uterine cavity upon deposition.

A healthy adult uterine cavity secretes 3–4 g of endometrial fluid every 4 h [8], so hydrogels intended for intrauterine application must be able to resist fluid flushing. To simulate the damaged endometrium, the endometrial tissues of pigs were scraped. Unlike the sodium hyaluronate (HA) hydrogel, which is clinically utilized for treating endometrial injury, LAT@L hydrogel adheres well to the injured endometrium even after 24 h of rinsing (Figure 2g). Lap shear testing shows that LAT@L hydrogel has an adhesion strength of about 8 kPa, substantially higher than that of HA hydrogel (0.5 kPa) (Figure 2h). SEM image reveals that a tight interface is formed between the injured endometrium and LAT@L hydrogel (Figure 2i). To better simulate the dynamic uterine environment, LAT@L hydrogel was injected into the damaged porcine endometrium and incubated in simulated uterine fluid at 37°C with gentle agitation. After 48 h, LAT@L hydrogel still adheres well to the injured endometrium (Figure S8). These results demonstrate that LAT@L hydrogel adheres effectively to the injured endometrium and exhibits resistance to mucus flushing.

**FIGURE 3:**
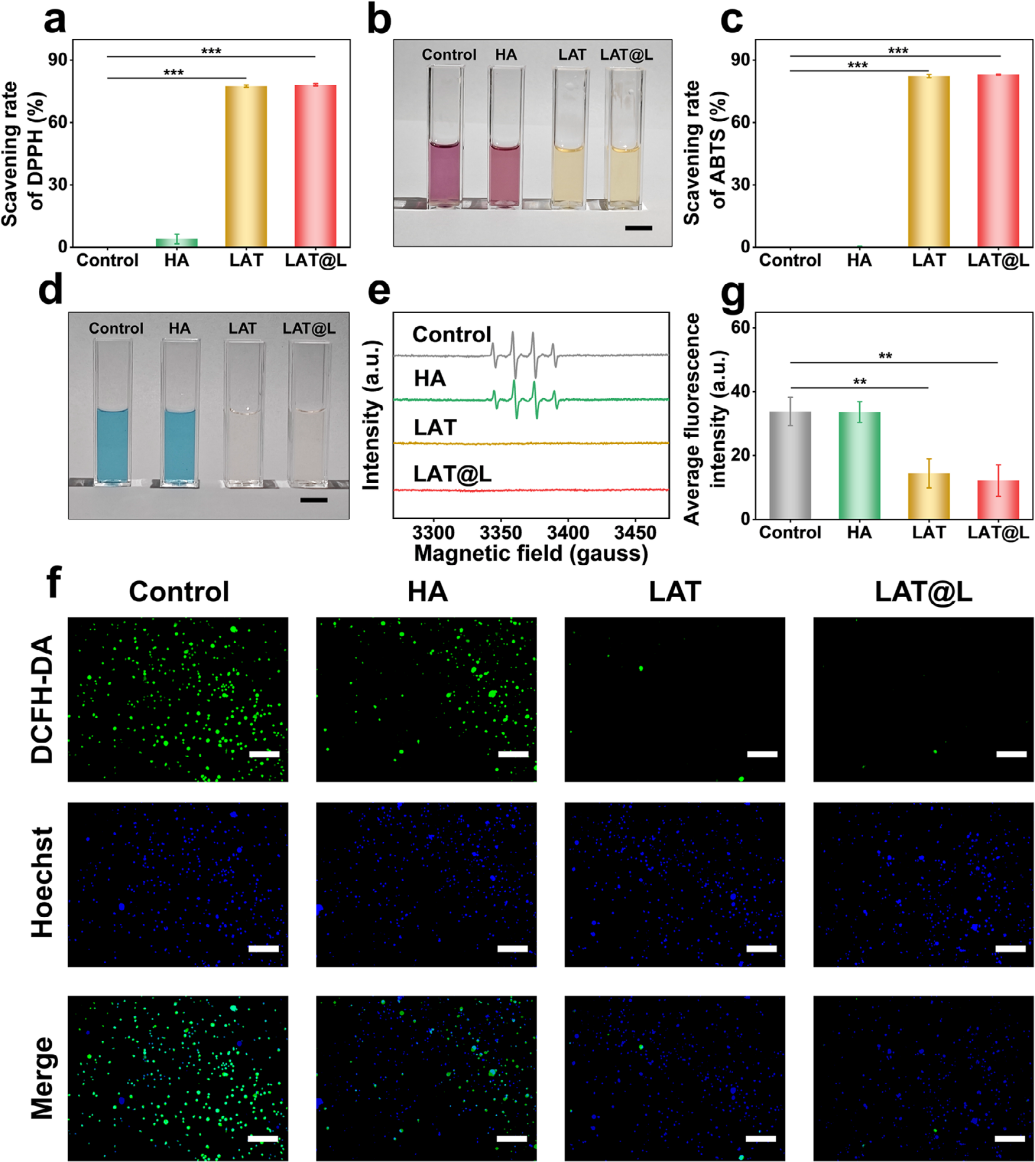
Antioxidant properties of LAT@L hydrogel. (a) DPPH• scavenging rates, (b) DPPH• solutions, (c) ABTS^+^• scavenging rates, and (d) ABTS^+^• solutions of the control, HA, LAT, and LAT@L groups (scale bars: 1 cm). (e) EPR spectra of DMPO‐•OH in the presence of different samples and TiO_2_ under UV irradiation. (f) Fluorescence images of L929 cells with different treatments and (g) corresponding fluorescence intensity statistics. The cells were stained with DCFH‐DA (green fluorescence) and Hoechst (blue fluorescence) (scale bars: 40 µm). All data are presented as mean ± SD (*n* = 3), and intergroup differences were evaluated using one‐way ANOVA followed by Tukey's post‐test. Statistical significance was defined as ^**^ *p* < 0.01, ^***^ *p* < 0.001.

Uterine injury is associated with disrupted blood flow, leading to localized hypoxia that subsequently activates circulating neutrophils and macrophages, resulting in a rapid release of free radicals [32, 33]. These free radicals facilitate the secretion of extracellular matrix and induce epithelial‐mesenchymal transition. Moreover, an overabundance of reactive oxygen species (ROS) can cause lipid peroxidation, alterations in protein structure, and DNA damage, thereby hindering the reparative processes of the uterus [34]. To evaluate the radical‐scavenging capacity of LAT@L hydrogel, we assessed its efficacy against free radicals of 2,2‐diphenyl‐1‐picrylhydrazyl (DPPH•), 2,2'‐azino‐bis (3‐ethylbenzothiazolin‐6‐sulfonic acid) (ABTS^+^•), and hydroxyl radicals (•OH) (Figure 3a–e and Figure S9). LAT@L hydrogel demonstrates effective scavenging of all tested radicals, showing significant antioxidant activity.

To further substantiate the in vitro antioxidant efficacy of LAT@L hydrogel for the treatment of endometrial injury, an oxidative microenvironment model was established by exposing L929 cells to hydrogen peroxide (H_2_O_2_). The intracellular ROS levels in L929 cells under various treatment conditions were evaluated using fluorescence microscopy. Cells were co‐stained with 2',7'‐dichlorodihydrofluorescein diacetate (DCFH‐DA), a fluorogenic probe for ROS detection, and Hoechst 33342, a cell‐permeant nuclear counterstain. The stimulation with H_2_O_2_ induces significant oxidative stress, resulting in elevated fluorescence intensities in the control group (Figure 3f,g). Conversely, the LAT@L group exhibits a substantial reduction in green fluorescence intensity, indicating that LAT@L hydrogel possesses a robust intracellular ROS scavenging capability and can mitigate oxidative stress in cells. Therefore, LAT@L hydrogel has the potential to mitigate fibrosis by efficiently neutralizing free radicals associated with oxidative stress. This antioxidant property not only contributes to the suppression of fibrosis but also aids in reducing endometrial tissue damage caused by oxidative stress, thereby promoting healing.

### 2.2 Biocompatibility, Sustained Uterine Retention and Flush Resistance of LAT@L Hydrogel

Considering that LAT hydrogel comes into direct contact with both the endometrium and *L. johnsonii*, its biocompatibility is crucial for the efficacy of microbial therapies and endometrial injury repair. In vitro cytotoxicity assessment shows that the number of L929 fibroblasts incubated with LAT hydrogel exhibits no significant decrease compared to the control group on days 1, 2, and 3 (Figure S10). To assess the impact of LAT hydrogel on *L. johnsonii* viability, the growth curve and live/dead staining of the *L. johnsonii* released from LAT@L hydrogel are compared with those of free bacteria, and no significant differences are observed at any tested time point (Figure S11). Additionally, LAT@L hydrogel was rinsed with PBS for 1 h to remove surface‐adhered bacteria, then cut into smaller pieces and incubated in 10 mL of PBS at 37°C for 10 min. The bacteria released from the hydrogel were subsequently plated for colony counting (Figure S12a,b). The result indicates that LAT hydrogel does not adversely affect *L. johnsonii* proliferation. Furthermore, the pH levels in the LAT@L hydrogel group are comparable to those in the free *L. johnsonii* group over 48 h (Figure S12c). These findings imply that LAT hydrogel's 3D network provides a favorable microenvironment with interconnected porous channels that facilitate nutrient and waste exchange, thereby preserving bacterial bioactivity.

**FIGURE 4:**
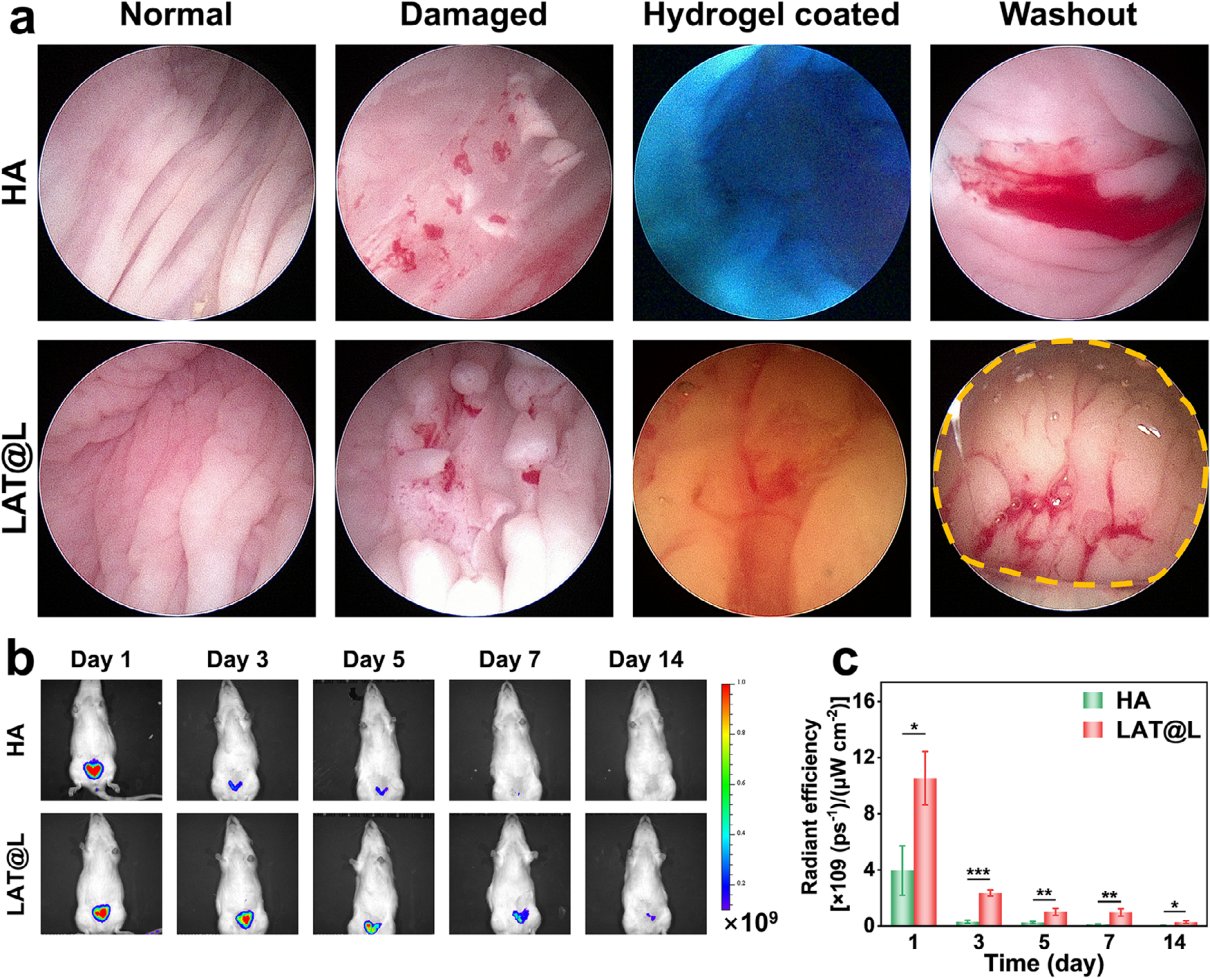
(a) The coverage and adhesion of methylene blue‐stained HA hydrogel and LAT@L hydrogel were observed by hysteroscopy on the injured porcine endometrial model. (b) Fluorescence images and (c) corresponding statistics of HA and LAT@L hydrogels in the uterus of rats for 1, 3, 5, 7, and 14 days (*n* = 3). All data are presented as mean ± SD (*n* = 3), and intergroup differences were evaluated using one‐way ANOVA followed by Tukey's post‐test. Statistical significance was defined as ^*^ *p* < 0.05, ^**^ *p* < 0.01, ^***^ *p* < 0.001.

The prolonged retention of LAT@L hydrogel is essential for establishing an adequate physical barrier and ensuring stable retention of *L. johnsonii*. In vitro studies have shown that LAT@L hydrogel can be retained for up to 14 days at 37°C (Figure S13). To more accurately replicate clinical procedures, hysteroscopic surgery was conducted on a porcine model. The endometrial tissue was intentionally damaged using hysteroscopic scissors, after which either LAT@L hydrogel or methylene blue‐stained HA hydrogel was administered. Unlike HA hydrogel, LAT@L hydrogel successfully adheres to the wound site and resists dislodgement by the distension fluid employed during hysteroscopy (Figure 4a). To evaluate LAT@L hydrogel retention in the uterine cavity, we monitored the fluorescence intensity of Cy5.5‐labeled LAT@L hydrogel on days 1, 3, 5, 7, and 14 post‐injection. Both groups have strong initial signals on day 1, but the fluorescence in the HA group diminishes more quickly within 7 days. LAT@L hydrogel maintains higher fluorescence throughout the observation, with a signal on day 14 (Figure 4b,c), indicating prolonged retention in the uterine cavity. These findings indicate that LAT@L hydrogel provides superior coverage of the uterine cavity and exhibits enhanced resistance to flushing compared to HA hydrogel.

### 2.3 In Vivo Analysis of the Effect of LAT@L Hydrogel on Endometrial Morphology

**FIGURE 5:**
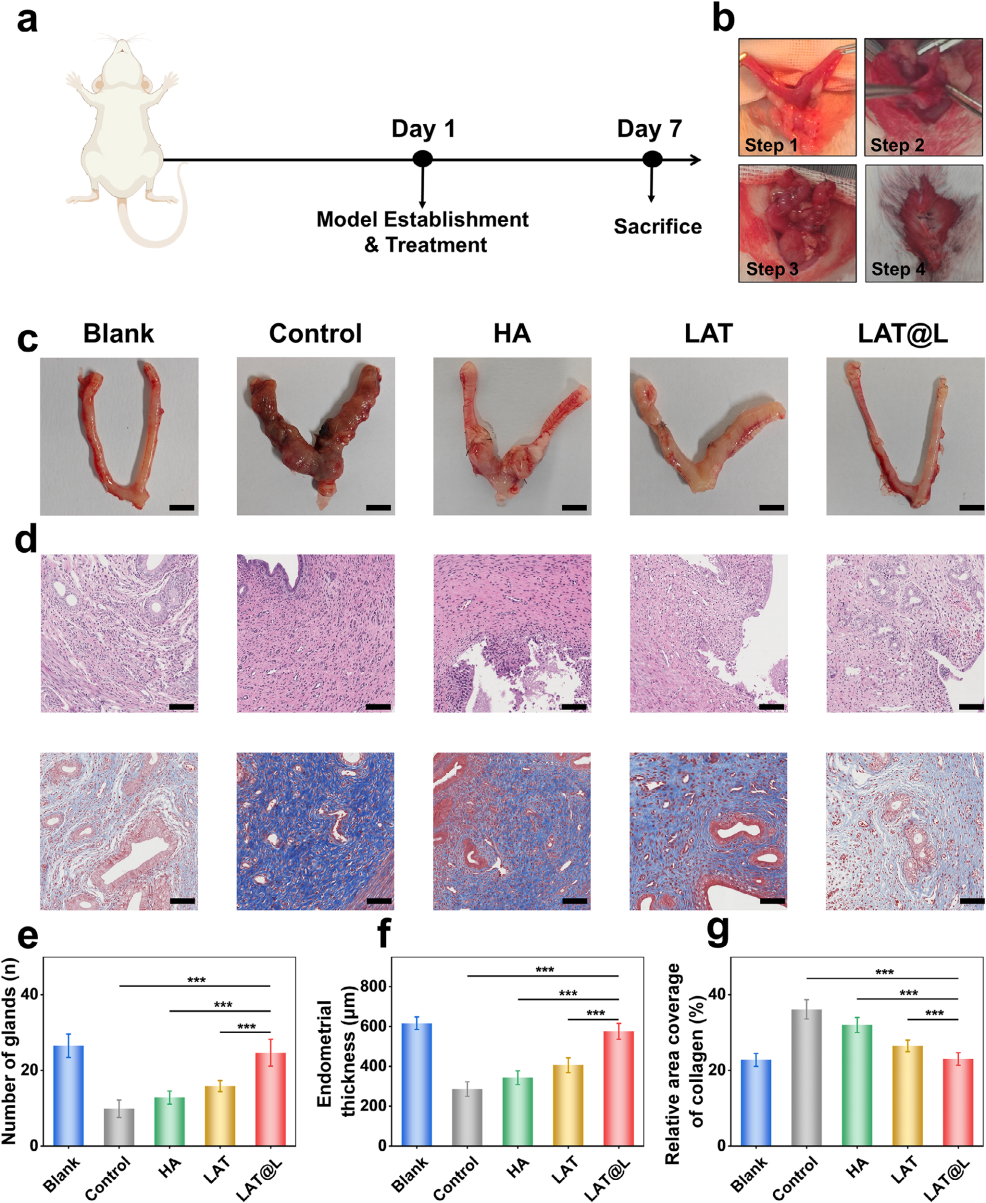
Effect of LAT@L hydrogel on endometrial morphology. (a) The total protocol of efficacy evaluation of different groups. (b) The procedures for the endometrial injury model of rats. (c) Photos of the uterus 7 days after different treatments (scale bars: 1 cm). (d) H&E and Masson's trichrome staining of the middle after treatment (scale bars: 20 µm). Quantitative analyses of (e) the number of endometrial glands, (f) the endometrial thickness, and (g) the relative area coverage of collagen. All data are presented as mean ± SD (*n* = 6), and intergroup differences were evaluated using one‐way ANOVA followed by Tukey's post‐test. Statistical significance was defined as ^***^ *p* < 0.001.

The pro‐healing effect of LAT@L hydrogel was evaluated in the rat model of endometrial injury (Figure 5a,b), which was established according to the procedures described in a previous study [35]. Following a one‐week acclimatization, the rats were randomly assigned to 5 groups: blank (normal rats with sham operation), control (rats with uterine injury surgery without treatment), HA, LAT, and LAT@L. All treatments were administered via direct intrauterine injection. After one week of treatment, the uterine tissue appearance in the LAT@L group closely resembles that of the blank group (Figure 5c). In contrast, the control group exhibits noticeable endometrial cavity fluid, intrauterine adhesions, and tissue deformation. The HA hydrogel group continues to display uterine swelling with significant hydrops. Compared to the control group, both the LAT and LAT@L groups demonstrate varying degrees of reduction in intrauterine fluid and adhesions, with the LAT@L hydrogel treatment showing the most substantial therapeutic effect.

The findings from hematoxylin and eosin (H&E) and Masson's trichrome staining (Figure 5d) demonstrate an increase in endometrial thickness across all groups. Specifically, LAT@L hydrogel treatment increases endometrial thickness from 285 µm in the control group to a maximum of 576 µm, achieving approximately 93.5% of the thickness observed in the blank group (Figure 5f). This underscores the substantial regenerative potential of LAT@L hydrogel. Additionally, the observed trend in gland number changes aligns with the increase in endometrial thickness (Figure 5e), further substantiating the hydrogel's promotive effect on endometrial regeneration. Masson's staining also reveals that the collagen structure in the LAT@L group closely resembles that of the uninjured endometrium. Compared to the LAT and HA groups, the LAT@L group shows a significant reduction in the fibrotic area within the endometrium (Figure 5g). These results suggest that LAT@L hydrogel possesses commendable pro‐healing properties.

### 2.4 Effect of LAT@L Hydrogel on Receptivity and Fertility of Rats

**FIGURE 6:**
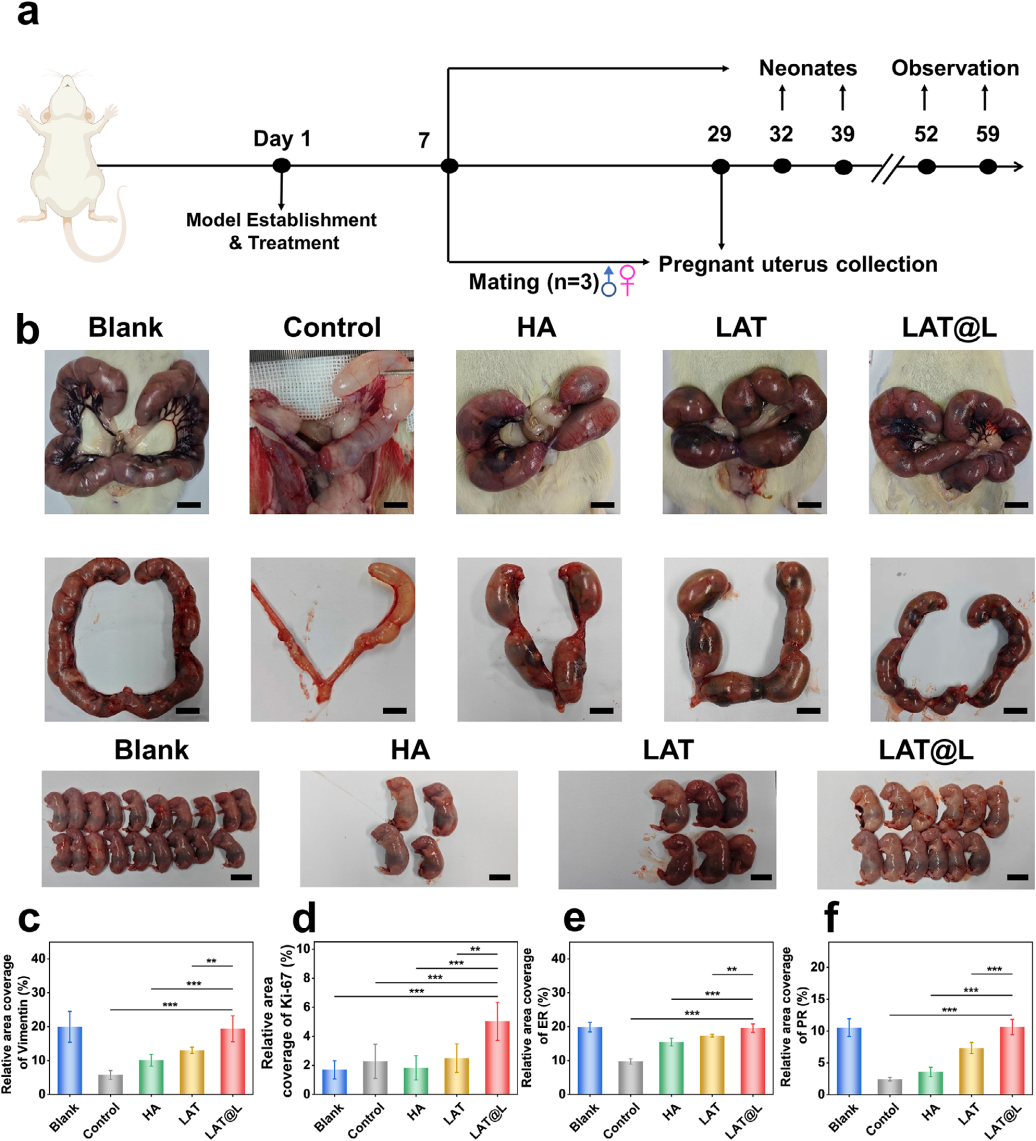
Effect of LAT@L hydrogel on fertility of rats and endometrial receptivity. (a) Experimental procedure. (b) Live birth outcomes in vivo of different groups (scale bars: 2 cm). Quantitative analysis of (c) Vimentin, (d) Ki‐67, (e) ER, and (f) PR. All data are presented as mean ± SD (*n* = 3), and intergroup differences were evaluated using one‐way ANOVA followed by Tukey's post‐test. Statistical significance was defined as ^**^ *p* < 0.01, ^***^ *p* < 0.001.

Embryonic implantation and fertility serve as essential indicators of uterine functionality. Therefore, we evaluated the reproductive performance of the treated rats by examining fertilized egg implantation, embryonic development, and live birth outcomes (Figure 6a). To evaluate the impact of LAT@L hydrogel on egg implantation, female rats were paired for mating 7 days post‐surgery. The rate of egg implantation was calculated by collecting the embryos at 29 days post‐surgery. The egg implantation outcome in the control group is zero, whereas the LAT@L group exhibits a obvious improvement (Figure 6b and Figure S14b). The appearance of neonates and body weight during 4 weeks were recorded (Figure S14a,d). No significant differences in appearance and weight are observed among the control, blank, LAT, and LAT@L groups. Regarding live births, no newborns are observed in the control group, whereas LAT@L hydrogel treatment increases the average number of neonates to approximately 11 (Figure S14c). Both embryo number and neonate health in the LAT@L group indicate normal endometrial function, suggesting the significant capacity of LAT@L hydrogel for endometrial receptivity and fertility.

Cell proliferation is a critical factor in the process of endometrial repair [36]. The immunohistochemistry (IHC) analysis of Vimentin shows that the area of endometrial stromal cells is reduced in the control group (Figure 6c and Figure S15), but it is recovered by LAT@L hydrogel treatment. Besides, the expression of Ki‐67 in the LAT@L group is significantly higher than that in the control group (Figure 6d and Figure S15), suggesting that the observed increase in endometrial thickness might be attributed to the proliferation of endometrial cells. Furthermore, to validate the restoration of endometrial tolerance and functionality after surgery, the IHC of estradiol receptor (ER) and progesterone receptor (PR) was performed on the uterus (Figure 6e,f and Figure S15). ER and PR, expressed in the nuclei of endometrial epithelial and stromal cells, are crucial for the regulation of reproductive processes, including menstruation and pregnancy [37, 38]. Given that the stromal and epithelial cells in the LAT@L group recover better, the expression levels of ER and PR are more similar to those of the blank group. Overall, the LAT@L group exhibits enhanced restoration of uterine tolerance and functionality, thereby facilitating normal conception and childbirth in subsequent stages.

**FIGURE 7:**
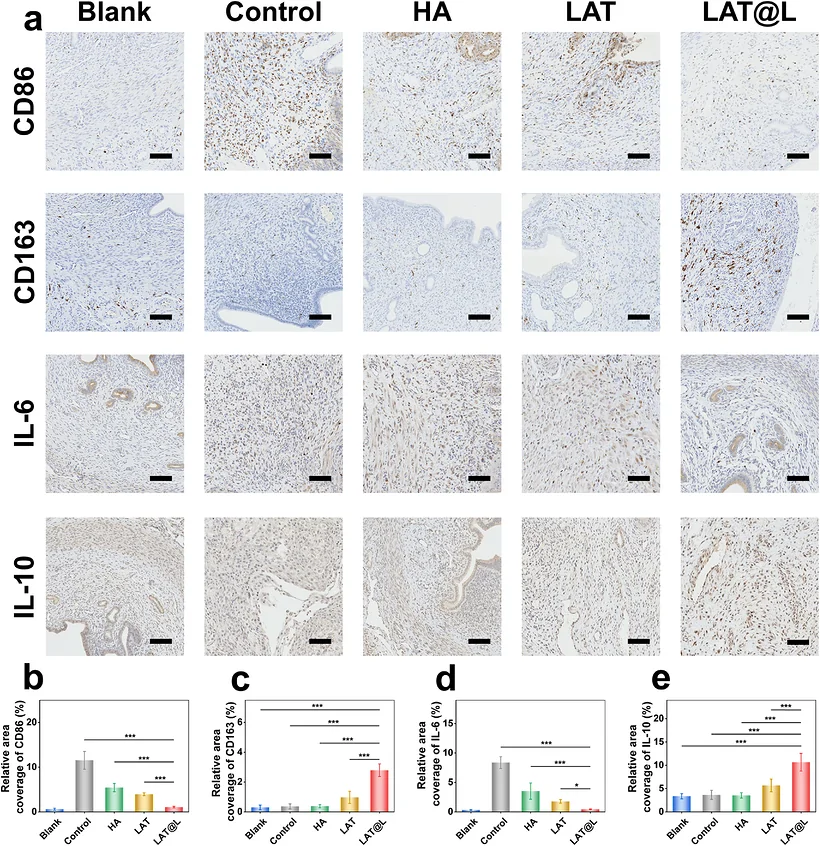
In vivo effects of various hydrogels on inflammation and macrophages. (a) Images of IHC staining of CD86, CD163, IL‐6, and IL‐10 for different groups (scale bars: 100 µm). Quantitative analysis of (b) CD86, (c) CD163, (d) IL‐6, and (e) IL‐10. All data are presented as mean ± SD (*n* = 6), and intergroup differences were evaluated using one‐way ANOVA followed by Tukey's post‐test. Statistical significance was defined as ^*^ *p* < 0.05, ^***^ *p* < 0.001.

To explore the treatment mechanism of LAT@L hydrogel for endometrial injury, IHC staining of M1 macrophage marker CD86 as well as M2 macrophage marker CD163 was performed to evaluate the immune regulatory effect of LAT@L hydrogel. The results demonstrate a significant reduction in CD86 expression and a marked increase in CD163 expression in the LAT@L group compared to other groups (Figure 7a–c). Additionally, there is a corresponding decrease in IL‐6 levels and an increase in IL‐10 levels (Figure 7a,d,e). These findings suggest that LAT@L hydrogel effectively induces M2 macrophage polarization and exerts an anti‐inflammatory effect during the repair of endometrial injury.

Ensuring the biosafety of LAT@L hydrogel is crucial for its application in endometrial injury therapy. To this end, body weight, H&E staining of major organs (heart, liver, spleen, lung, and kidney), and serum chemistry indices were measured (Figure S16). Throughout the study, no significant weight loss is detected in any groups (Figure S16b). Additionally, H&E staining indicates no discernible adverse effects on the major organs across all groups (Figure S16c) and no detrimental impact on uterine tissue (Figure S16d). Serum analyses reveal no significant changes in cardiac injury markers (creatine kinase isoenzyme), liver function markers (alanine aminotransferase, aspartate aminotransferase, total bilirubin, and albumin), and renal function markers (blood urea nitrogen and creatinine) (Figure S16e–g). Collectively, these findings underscore the excellent in vivo safety profile of LAT@L hydrogel, highlighting its potential suitability for endometrial injury treatment.

### 2.5 Modulatory Effect on Uterine Microbiome

**FIGURE 8:**
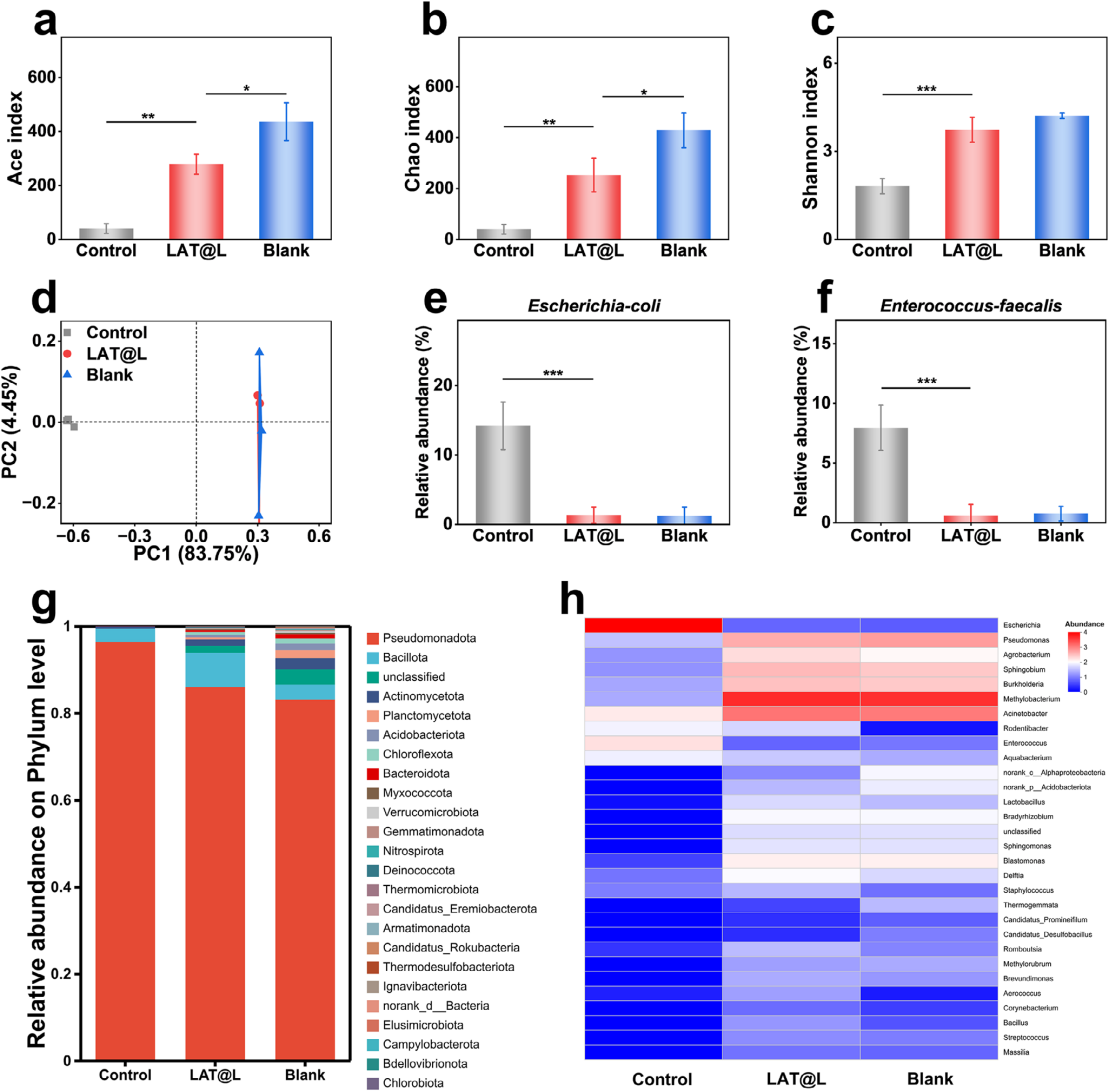
*α*‐diversity analyses of uterine microbiome illustrated by (a) Ace, (b) Chao index, and (c) Shannon index. (d) *β*‐diversity analysis of the uterine microbiome demonstrated by the PCoA plot in different groups on day 7. Relative genus abundance of (e) *Escherichia‐coli* and (f) *Enterococcus‐faecalis* in different groups on day 7. (g) Relative abundance of uterine microbiome at the phylum level. (h) Heatmap depicting the relative abundance of uterine microbiome at the genus level. All data are presented as mean ± SD (*n* = 3), and intergroup differences were evaluated using one‐way ANOVA followed by Tukey's post‐test. Statistical significance was defined as ^*^ *p* < 0.05, ^**^ *p* < 0.01, ^***^ *p* < 0.001.

Endometrial injury is linked to dysbiosis of the uterine microbiota, which disrupts immune homeostasis within the uterine cavity and leads to infertility [39, 40]. To investigate the effect of LAT@L hydrogel treatment on uterine microbiota composition, we used 16S rRNA sequencing technology to analyze uterine swabs collected from each group on day 7. Analyses of *α*‐diversity, employing Ace, Chao, and Shannon indices (Figure 8a–c), indicate that the LAT@L group shows a significant improvement in microbial diversity compared to the control group, with diversity levels closely approximating those of the blank group. Principal coordinates analysis (PCoA) of *β*‐diversity reveals (Figure 8d) that the LAT@L group exhibits a high degree of similarity to the blank group, whereas there is a distinct separation between the control group and the blank group. Furthermore, Venn diagram analysis suggests that LAT@L hydrogel treatment enhances microbial richness (Figure S17).

To comprehensively assess alterations in the microbial community, we quantified taxonomic abundance at both the phylum and genus levels. At the phylum level, the LAT@L group exhibits a microbial profile similar to that of the blank group (Figure 8g), with a significantly increased relative abundance of phylum Bacillota compared to the control group. Analysis at the genus level (Figure 8h) indicates that the LAT@L group substantially enhances the microbiome structure, notably decreasing the relative abundance of harmful genera such as *Escherichia‐coli* (Figure 8e) and *Enterococcus‐faecalis* (Figure 8f), which are known to exacerbate chronic endometritis through toxin production, metabolic dysregulation, and pathogenic synergism [41, 42, 43, 44]. These results collectively indicate that the treatment with LAT@L hydrogel effectively reestablishes uterine ecological balance within the endometrial injury microenvironment.

## 3 Conclusion

In summary, we have successfully developed a multifunctional adhesive LAT@L hydrogel by facilely integrating three natural medicinal molecules with *L. johnsonii*, which exhibits extended intrauterine retention, significant antioxidant properties, and the ability to synergistically modulate the microenvironment. The stable network structure of LAT@L hydrogel is spontaneously constituted by covalent bonds, hydrogen bonds, and electrostatic interactions among LA, TA, and Arg. The presence of active disulfide bonds, carboxyl groups, and polyphenolic groups of LAT@L hydrogel facilitates robust interfacial adhesion to tissue surfaces. The excellent cohesion and adhesion resulting from these synergistic interactions endow LAT@L hydrogel with a long‐term retention capacity in the uterine cavity exceeding 14 days. Meanwhile, LAT@L hydrogel can effectively scavenge ROS to provide cellular protection against oxidative stress and modulate the uterine microbiota to reestablish ecological balance, thereby creating a pro‐regenerative microenvironment. Consequently, LAT@L hydrogel markedly enhances endometrial repair and facilitates fertility recovery in a rat model of endometrial injury. We anticipate that our work will advance the development of innovative injectable hydrogels and serve as a model for the design of sophisticated biomaterials for endometrial repair.

## Author Contributions

**Qiuxian Xie**: validation, investigation. **Hui Zhou**: conceptualization, methodology, software, investigation, validation, writing – original draft. **Yi Liang**: data curation, software. **Yifei Li**: conceptualization, methodology, investigation, validation, writing – original draft. **Yu Zhang**: conceptualization, methodology, supervision, resources, project administration, visualization, funding acquisition, writing – original draft, writing – review and editing. **Lifa Chen**: conceptualization, methodology, software, data curation, investigation, validation, formal analysis, writing – original draft. **Youchen Tang**: conceptualization, supervision, visualization, project administration, resources, writing – original draft, writing – review and editing. **Yifan Zhang**: validation, investigation. **Huixia Ye**: conceptualization, methodology, supervision, funding acquisition, visualization, project administration, resources, writing – original draft, writing – review and editing. **Huiyu Huang**: software, data curation.

## Conflicts of Interest

The authors declare no conflicts of interest.

## Supporting information




**Supporting File**: advs77229‐sup‐0001‐SuppMat.docx.

## Data Availability

The data that support the findings of this study are available from the corresponding author upon reasonable request.
