# Supplementary material for: Lactobacillus‐Loaded Bioadhesive Hydrogel with Prolonged Retention and Microenvironmental Remodeling for Endometrial Repair and Fertility Restoration
Source: Adv Sci (Weinh). 2026 Aug 21:e77229. Online ahead of print. doi: 10.1002/advs.77229 (PMC13496152; doi:10.1002/advs.77229)
Supplement: Supplementary file 1 [file ADVS-9999-e77229-s001.docx]

**Supporting Information**

## Lactobacillus-Loaded Bioadhesive Hydrogel with Prolonged Retention and Microenvironmental Remodeling for Endometrial Repair and Fertility Restoration

Lifa Chen^1^, Hui Zhou^2^, Yifei Li^3^, Yi Liang^1^, Yifan Zhang^1^, Huiyu Huang^1^, Qiuxian Xie^4^, Youchen Tang^5,*^, Huixia Ye^1,*^, and Yu Zhang^1,*^

^1^Department of Gynecology, The Third Affiliated Hospital, Sun Yat-sen University, Guangzhou 510630, China

^2^Key Laboratory for Polymeric Composite & Functional Materials of Ministry of Education, School of Chemistry, Sun Yat-sen University, Guangzhou 510006, China

^3^Colorectal Surgery Unit III, Guangdong Institute of Gastroenterology, Biomedical Innovation Center, Key Laboratory of Human Microbiome and Chronic Diseases, Sun Yat-sen University, Ministry of Education, Guangdong Provincial Key Laboratory of Colorectal and Pelvic Floor Diseases, The Sixth Affiliated Hospital, Sun Yat-sen University, Guangzhou 510655, China

^4^Department of Gynecology, Chaozhou Central Hospital, Chaozhou 521021, China

^5^The Eighth Affiliated Hospital, Sun Yat-sen University, Shenzhen 518033, China

**Correspondence:** Youchen Tang (tangych7@mail.sysu.edu.cn), Huixia Ye (yehx5@mail.sysu.edu.cn), and Yu Zhang (zhangyu6@mail.sysu.edu.cn)

**Experimental Section**

**Materials**

L-arginine (Arg, AR), tannic acid (TA, Mw = 1701.2 Da), *α*-lipoic acid (LA, 99%), 2,2-diphenyl-1-picrylhydrazyl (DPPH) and Cy5.5-NHS ester were purchased from Shanghai Macklin Biochemical Technology Co., Ltd. (China). Potassium persulfate, ABTS^+^, and 5,5-dimethyl-1-pyrroline-*N*-oxide (DMPO) were purchased from Shanghai Aladdin Biochemical Technology Co., Ltd. (China). Cross-linked sodium hyaluronate (HA) hydrogel for the uterine cavity, trade name Gong' ankang, was purchased from Changzhou Bioregen Biomedical Co., Ltd. (China). Phosphate-buffered saline (PBS) buffer (pH = 7.2 ~ 7.4) was purchased from Wuhan Servicebio Technology Co., Ltd. (China). Man-Rogosa-Sharpe (MRS) medium was obtained from Guangdong Huankai Microbial Science & Technology Co., Ltd. (China). The standard fibroblastic cell line L929 was sourced from the Procell Life Science & Technology Co., Ltd. (China). Cell counting kit-8 (CCK-8), calcein acetoxymethyl ester (Calcein AM)/propidium iodide (PI) double staining kit, DMAO/PI and Hoechst 33342 were procured from Beyotime Biotechnology Co., Ltd. (China). 2′,7′-dichlorodihydrofluorescein diacetate (DCFH-DA) was purchased from Beijing Solarbio Science & Technology Co., Ltd. (China). Dulbecco's Modified Eagle medium (DMEM), fetal bovine serum (FBS), penicillin-streptomycin solution (PSS), and trypsin-ethylenediaminetetraacetic acid (EDTA) were purchased from Gibco-BRL Life Technologies, Inc. (USA). *Lactobacillus johnsonii* (*L. johnsonii*) was obtained from the Jiangnan University (China). Hoechst 33342 was procured from Beyotime Biotechnology Co., Ltd. (China). DCFH-DA was purchased from Beijing Solarbio Science & Technology Co., Ltd. (China). All solvents and chemicals were purchased from commercial sources and used without further purification.

**Synthesis of LAT and LAT@L Hydrogels**

*L. johnsonii* was cultured in an anaerobic chamber (COY-8300400, COY, USA). Solution A was prepared by dissolving 150 mg of Arg and 300 mg of LA in 1 mL of deionized water. For the synthesis of LAT hydrogel, solution A was added to 50 mg of TA and stirred vigorously at 1000 rpm for 10 min. To prepare LAT@L hydrogel, 1.0 mL of solution A was combined with 0.1 mL of PBS containing *L. johnsonii* (10⁷ CFU mL⁻¹), and this mixture was then vigorously stirred with 50 mg of TA at 1000 rpm for 10 min to yield LAT@L hydrogel.

**Material Characterization**

The microstructure of LAT@L hydrogel was observed using scanning electron microscopy (SEM, S-4800, Hitachi, Japan). Prior to observation, the lyophilized LAT@L hydrogel underwent platinum sputter coating (10 mA, 50 s) twice for enhanced imaging. Fourier-transform infrared (FTIR) spectra were recorded on an FTIR spectrometer (Nexus 670, Thermo Scientific, USA). Chemical structures were characterized by using an X-ray photoelectron spectrometer (XPS, K-Alpha, Thermo Scientific, USA). Raman spectra were studied on a Raman spectrometer (alpha300R, WITec, Germany). X-ray diffraction (XRD) patterns were investigated on an X-ray diffractometer (D-MAX 2200 VPC, Rigaku, Japan). Before conducting FTIR, XRD, and XPS analyses, the samples were freeze-dried.

**Rheological Characterization**

Rheological measurements, encompassing shear viscosity, shear strain, G', and G'', were conducted utilizing a rotational rheometer (Kinexus Pro+, Malvern, UK). In accordance with the instrument's operational protocol, LAT@L hydrogel was positioned on the rotating platform, with all experiments performed at a constant temperature of 25°C. During oscillatory frequency scanning, a fixed strain of 1% was applied over a frequency range of 0.1 to 10.0 Hz. Shear viscosity profiles were derived through yield stress testing. Amplitude scanning, ranging from 0.1% to 1000%, were performed at a constant frequency of 0.1 Hz. Cyclic amplitude scanning tests followed a specific sequence: initially, the hydrogel was subjected to a 1% strain; subsequently, the strain was increased from 1% to 400% and maintained for 1 min to completely disrupt the gel network; finally, the strain was decreased from 400% back to 1% and held for 5 min to assess the recovery of the gel network.

**Release of *L. johnsonii* In Vitro**

Initially, 1 mL of LAT@L hydrogel was immersed in 4 mL of sterile PBS buffer. The samples were incubated with an oscillating speed of 120 rpm at 37℃. At specific time intervals (0, 6, 12, 24, 36, 48, 60, and 72 h), the incubated PBS solution was diluted in a 10-fold series to 10^−2^ or 10^−3^. MRS agar plates were cultivated for 48 h at 37°C. Each group conducted three parallel experiments. The release rate is defined as follows: Release rate = (*N_t_* / N) ×100%, where N and *N_t_* represent the total number of colonies and the number of colonies at different times, respectively.

**Assessment of *L. johnsonii* Viability**

An aliquot of 0.1 mL of LAT@L hydrogel was submerged in 1 mL of MRS medium. The pH of the resulting solution was assessed at four distinct time intervals (0, 12, 24, and 48 h) utilizing a pH meter (Sharp tip pH meter, 8694 AZ EB, Taiwan, China). LAT@L hydrogel samples were collected at 0, 24, and 48 h, weighed in glass bottles, and rinsed with sterile PBS for 1 h, with the solution changed hourly, to remove surface bacteria. The hydrogels were then cut into pieces, mixed with 10 mL of PBS, and incubated at 37℃ for 10 min. The resulting solution was spread on agar plates for bacterial counting.

1 mL of LAT@L hydrogel was immersed in 4 mL of sterile PBS buffer. The samples were incubated at an oscillating speed of 120 rpm at 37℃. After 3 days, the incubated PBS solution was centrifuged at 8000 rpm for 3 min and resuspended in MRS solution. Bacterial growth was monitored by measuring OD600 at specific time intervals during incubation at 37℃. Pure MRS served as the blank reference. Additionally, at specific time intervals, the incubated PBS solution was stained with DMAO and PI. The number of live/dead bacteria was observed under a fluorescence microscope (DP80, Olympus, Japan).

**In Vitro Retention Assessment**

To assess hydrogel retention on the endometrial surface, pig endometrial tissues were excised and scraped. Hydrogel was injected into both the endometrial tissues. After PBS rinsing, retention was recorded, and the adhesion interface was analyzed using a SEM. To better simulate the dynamic physiological conditions within the uterine cavity, LAT@L hydrogel was injected into a damaged porcine endometrium and then immersed in simulated uterine fluid (ingredients as shown in Table S1) at 37℃ with a shake frequency of 2 times per min. Afterwards, the residual hydrogel on the damaged porcine endometrium was captured with a camera.

**In Vitro Compatibility Assessment**

1.0 mL of the hydrogel sample was mixed with 5.0 mL of DMEM solution and placed in a 37°C incubator for 24 h. Then, the culture medium was obtained using a 0.22 μm bacterial filter for further use. 3 × 10^3^ L929 fibroblasts were seeded in 96-well plates with 100 μL of DMEM solution, 10% FBS, and 1% penicillin−streptomycin, and incubated overnight under 5% CO_2_ at 37°C. 100 μL of the culture medium was added to each well of the test group, while 100 μL of DMEM solution was added to each well of the control group. At 24, 48, and 72 h after incubation, 100 μL of 10% CCK-8 assay solution was added to each well of both groups under dark conditions. After 1 h, cell proliferation was determined by measuring the fluorescence absorbance at a wavelength of 450 nm. Optical density values were measured using a multifunctional measuring instrument (Varioskan LUX, Thermo Fisher Scientific, USA). Additionally, 100 μL of calcein-AM solution (2 μg mL^−1^) was added to each well of both groups under dark conditions. After 30 min, stained L929 fibroblast cells were observed under a fluorescence microscope (DP80, Olympus, Japan).

**DPPH Radical Scavenging Assay**

A 100 μM DPPH• solution in ethanol was prepared, and 100 mg of either HA, LAT, or LAT@L hydrogel was added to 3 mL of this solution. The mixtures were incubated at 37°C with shaking at 120 rpm for 1 h in the dark. Afterward, 200 μL of each was diluted, and UV-Vis absorption spectra from 450–600 nm was recorded to observe changes in the DPPH• signal, with absorbance at 517 nm measured using a multifunctional measuring instrument. The DPPH• radical scavenging rate was calculated according to the following formula: Scavenging rate = [(A_0_ – *A_1_*) / A_0_] ×100%, where A_0_ is the absorbance of the blank and *A_1_* is the absorbance of the sample.

**ABTS Radical Scavenging Assay**

A stock solution of ABTS⁺• radical was prepared by mixing 25 mL of 7.4 mM ABTS aqueous solution with 25 mL of 2.45 mM potassium persulfate solution. The mixture was incubated in the dark at 25°C for 12 h. The working solution was prepared by diluting 50 μL of the ABTS⁺• stock solution with 3 mL of distilled water. 100 mg of HA hydrogel, LAT hydrogel, or LAT@L hydrogel was added to 3 mL of the diluted ABTS⁺• working solution and incubated in the dark at 25°C for 20 min. Afterward, 200 μL of each was diluted, and UV-Vis absorption spectra from 600–900 nm were recorded to observe changes in the ABTS^+^• signal, with absorbance at 734 nm measured using a multifunctional measuring instrument. The ABTS⁺• scavenging activity was calculated using the formula: Scavenging rate = [(A_0_ – *A_1_*) / A_0_] ×100%, where A_0_ represents the absorbance of the ABTS^+^• working solution without sample, and *A_1_* represents the absorbance after addition of the hydrogel sample.

**Hydroxyl Radical Scavenging Assay**

Electron paramagnetic resonance (EPR) spectra were obtained using an EPR spectrometer (A300, Bruker, Germany). EPR experiment was carried out to confirm the •OH-scavenging activity of LAT@L hydrogel by using a TiO_2_/UV system to produce •OH. LAT@L hydrogel was added to 100 *µ*L of titanium dioxide dispersion (10 mg mL^−1^) followed by addition of 200 µL of DMPO. After 5 min exposure to UV-light (365 nm), EPR spectra were recorded.

**Intracellular ROS Detection**

After seeding L929 cells at a density of 1×10^4^ cells per well in 6‑well plates and culturing for 48 h, the cells were divided into four groups for co‑culture: (1) H_2_O_2_ treatment group, (2) HA + H_2_O_2_ group, (3) LAT + H_2_O_2_ group, and (4) LAT@L + H_2_O_2_ group. Following 2 h of co‑culture, the cells were stained sequentially with diluted DCFH‑DA for 25 min and Hoechst 33342 for 5 min. Green fluorescence (indicating intracellular ROS levels) and blue fluorescence (indicating live cell nuclei) were observed and captured using a fluorescence microscope (DP80, Olympus, Japan).

**In Vivo Retention Assessment of LAT@L Hydrogel**

A total of 0.5 mg of Cy5.5-NHS ester was mixed with 3.0 mL of hydrogel sample. Then, 400 µL of the mixture was injected into the uterine horns of a rat models (*n* = 3). Whole‑body fluorescence images of the rats were acquired using a small‑animal in vivo imaging system (IVIS) on days 1, 3, 5, 7, and 14 post‑injection. The corresponding fluorescence intensity was quantified using the instrument’s software to evaluate the retention of the hydrogel samples.

**Performance of LAT@L Hydrogel in a Pig Model**

A white pig (body weight 35−40 kg) was provided by Guangzhou Yinshe Medical Technology Co., Ltd. All experimental procedures were approved by the Institutional Animal Care and Use Committee of Guangzhou Yinshe Medical Technology Co., Ltd. (China) and conducted in strict accordance with the *Guide for the Care and Use of Laboratory Animals*. The pig was fasted for two days prior to the experiment, with free access to water. After anesthesia induced by xylazine hydrochloride and propofol, tracheal intubation was performed, and oxygen was continuously supplied at a flow rate of 2 L min^−1^. A hysteroscope was inserted into the uterine cavity for observation, followed by disruption of the endometrial basal layer. Subsequently, 3.0 mL of LAT@L hydrogel or methylene blue‑stained HA hydrogel was injected. One minute after injection, the uterine cavity was inspected, and the injured site was flushed with distention fluid. The entire procedure was documented, and the acquired images were analyzed for further evaluation.

**Establishment of the Rat Endometrial Injury Model and Treatment**

After the rats were anaesthetized with isoflurane (2%−3%, inhalation), the uterus was exposed using a lower abdominal midline incision. A 2 cm wound was made on the uterus. The endometrium was scraped using a T10 scalpel blade until the surface of the uterus became rough and bleeding occurred. After the uterus was washed with normal saline, it was sutured using 6–0 absorbable sutures. For treatment, the rats were randomly divided into five groups: blank (normal rats with sham operation), control (rats with uterine injury surgery without treatment), HA, LAT, and LAT@L. All treatments were administered once by direct injection into the uterus after the damage was induced. The skin and muscularis were stitched using 3–0 non-absorbable sutures. Meloxicam injection was administered following the surgery to reduce pain, and lidocaine was applied to the incision site. Animals that received same treatments were placed in the same cage. To ensure the survival of the rats after surgery, several measures were taken, including postoperative infrared radiation to maintain body temperature, feeding glucose saline to supplement water and energy, and timely replacement of bleeding to prevent wound infection.

**Histological Examination and Immunohistochemistry Staining**

A 0.5 cm length of uterine tissue was obtained at the site 0.5 cm above the surgical incision of each uterine branch, which was preserved in 4% paraformaldehyde solution for 24 h before being sent to Wuhan Servicebio Technology Co., Ltd. (China) for staining. The following staining protocol involved the application of H&E staining, Masson trichrome staining, and immunohistochemistry staining.

**In Vivo Toxicity Evaluation**

In vivo toxicities of LAT@L hydrogel were evaluated through pathological analysis of major organs in rat. After 7 days, the rats were sacrificed, and body weight, H&E staining of the main organs (heart, lung, spleen, liver, and kidney), and serum chemistry indices were performed to evaluate biological safety.

**Microbiota 16S Ribosomal RNA Gene Sequencing**

The 16S ribosomal RNA gene sequencing and analysis were conducted at Shanghai Majorbio Bio-Pharm Technology Co. Ltd. (China). First, total bacterial DNA was extracted from uterine swab of rats after various treatments. The hypervariable V3V4 regions of the bacterial 16S ribosomal RNA gene was amplified with primers 27F1492R and the obtained sequences were detected on the Illumina HiSeq platform. Data analysis was performed. The alpha diversity was analyzed to examine the richness and diversity of species in individual samples with Ace, Chao and Shannon indices, while beta diversity was analyzed with PCoA. Moreover, the species classification was obtained on the basis of the sequence composition of the ASV. Microbial composition was analyzed in phylum and genus levels.

**Animal Ethics Statement**

A white pig (body weight 35−40 kg) was provided by Guangzhou Yinshe Medical Technology Co., Ltd. (China). All experimental procedures were approved by the Institutional Animal Care and Use Committee of Guangzhou Yinshe Medical Technology Co., Ltd. (China) and conducted in strict accordance with the *Guide for the Care and Use of Laboratory Animals* (SS-2025-ZSL GQJ 1). Experimental rats were purchased from and bred at the Animal Center of Sun Yat-sen University (China). Animal experiments were reviewed and approved by the Institutional Animal Care and Use Committee (IACUC) at Sun Yat-Sen University (China, license number: SYSU-IACUC-2025-002904).

**Statistical Analysis**

Statistical analyses were performed using Origin 2026 software (Origin Lab Incorporation, Northampton, USA). Data were expressed as mean ± standard deviation (SD). Statistical differences between groups were determined by one‑way analysis of variance (ANOVA). Statistical significance indicated as **p* < 0.05, ***p* < 0.01, and ****p* < 0.001.

**Figure S1.** Possible interactions for the formation of LAT hydrogel.

**Figure S2.** Mixing different components at same mass ratio. In the absence of TA, the system results in the formation of a viscous liquid without gelation; without Arg, the solubility of LA is reduced, leading to the formation of precipitates. These observations underscore the critical role of the synergistic interaction among the three components in the construction of the hydrogel network.

**Figure S3.** a) XPS full spectrum of LAT hydrogel. b) N1s and c) C1s high-resolution scanning spectra of LAT. d) S2p and e) C1s high-resolution scanning spectra of LA. f) N1s high-resolution scanning spectrum of Arg.

**Figure S4.** SEM image of LAT@L hydrogel.

**Figure S5.** Release of *L. johnsonii* of LAT@L hydrogel in vitro. a) Colonies of *L. johnsonii* on MRS agar plates at specific time intervals. b) Cumulative release curve of *L. johnsonii* from LAT@L hydrogel. All data are presented as mean ± SD (*n* = 3).

**Figure S6.** Rheology properties of LAT@L hydrogel. a) Strain amplitude scanning curve at a fixed frequency of 0.1 Hz and b) shear viscosity-stress curve of LAT@L hydrogel.

**Figure S7.** a) In situ self-healing process of LAT@L hydrogel. b) Digital photos showing injectability for LAT@L hydrogel (scale bar: 1 cm).

**Figure S8.** Digital photos of LAT@L hydrogel on damaged pig endometrium after dynamic shaking in simulated uterine fluid at 37°C (scale bars: 1 cm).

**Figure S9.** UV-vis detection of the scavenging ability of a) DPPH• and b) ABTS^+^• by HA, LAT, and LAT@L hydrogels.

**Figure S10.** a) In vitro fluorescence images (scale bars: 40 μm) and b) CCK-8 assay of L929 fibroblasts co-cultured after 1, 2, and 3 days with the control and LAT hydrogel groups. The data are presented as mean ± SD (*n* = 3), and intergroup differences were evaluated using one-way ANOVA followed by Tukey’s post-test.

**Figure S11**. a) Growth curves of *L. johnsonii* in MRS and LAT hydrogel. b) Live/dead bacterial staining images of LAT@L hydrogel at day 0 and day 3 (scale bars, 400 μm). All data are presented as mean ± SD (*n* = 3), and intergroup differences were evaluated using one-way ANOVA followed by Tukey’s post-test.

**Figure S12.** a) Colonies of *L. johnsonii* on MRS agar plates after 0, 24, and 48 h incubation in LAT hydrogel. b) Colonies count statistics of *L. johnsonii*. All data are the mean ± SD (*n* = 3). c) pH values of MRS medium measured over 48 h for the control (free *L. johnsonii*) and LAT@L hydrogel groups.

**Figure S13.** Mass retention of LAT@L hydrogel in vitro.

**Figure S14.** a) Live birth outcomes *in vivo* of different treatment groups (scale bars: 1 cm). Number of b) fetuses and c) neonates in different treatment groups. d) Neonate weight in different treatment groups. All data are presented as mean ± SD (*n* = 3), and intergroup differences were evaluated using one-way ANOVA followed by Tukey's post-test. Statistical significance was defined as **p* < 0.05, ***p* < 0.01.

**Figure S15.** Images of immunohistochemical staining of Vimentin, Ki-67, ER, and PR for different groups after rat endometrial injury (scale bars: 100 μm).

**Figure S16.** Biological safety evaluation of LAT@L hydrogel. a) Procedure of the biosafety analysis. b) Body weight of rats. c) H&E staining of the main organs (scale bars: 100 μm). d) H&E staining of uterus tissue of rats for biological safety analysis (left scale bars:100 μm; right scale bars:30 μm). e-g) Serum chemistry indexes. All data are presented as mean ± SD (*n* = 3), and intergroup differences were evaluated using one-way ANOVA followed by Tukey’s post-test.

**Figure S17.** Venn diagram of the control, blank, and LAT@L groups.

**Table S1.** Composition of simulated uterine fluid.

| **Component** | **Concentration (g L^-1^)** |
| --- | --- |
| NaCl | 4.97 |
| KCl | 0.224 |
| CaCl_2_ | 0.167 |
| NaHCO_3_ | 0.25 |
| Glucose | 0.5 |
| NaH_2_PO_4_·2H_2_O | 0.072 |

Note: After preparation, adjust the pH to 7.0 using HCl or NaOH.
